# An Economic Framework of Microbial Trade

**DOI:** 10.1371/journal.pone.0132907

**Published:** 2015-07-29

**Authors:** Joshua Tasoff, Michael T. Mee, Harris H. Wang

**Affiliations:** 1 Department of Economics, Claremont Graduate University, Claremont, California, United States of America; 2 Department of Biomedical Engineering, Boston University, Boston, Massachusetts, United States of America; 3 Department of Systems Biology, Columbia University, New York, New York, United States of America; Cinvestav-Merida, MEXICO

## Abstract

A large fraction of microbial life on earth exists in complex communities where metabolic exchange is vital. Microbes trade essential resources to promote their own growth in an analogous way to countries that exchange goods in modern economic markets. Inspired by these similarities, we developed a framework based on general equilibrium theory (GET) from economics to predict the population dynamics of trading microbial communities. Our biotic GET (BGET) model provides an a priori theory of the growth benefits of microbial trade, yielding several novel insights relevant to understanding microbial ecology and engineering synthetic communities. We find that the economic concept of comparative advantage is a necessary condition for mutualistic trade. Our model suggests that microbial communities can grow faster when species are unable to produce essential resources that are obtained through trade, thereby promoting metabolic specialization and increased intercellular exchange. Furthermore, we find that species engaged in trade exhibit a fundamental tradeoff between growth rate and relative population abundance, and that different environments that put greater pressure on group selection versus individual selection will promote varying strategies along this growth-abundance spectrum. We experimentally tested this tradeoff using a synthetic consortium of *Escherichia coli* cells and found the results match the predictions of the model. This framework provides a foundation to study natural and engineered microbial communities through a new lens based on economic theories developed over the past century.

## Introduction

Metabolic exchange is a process by which microbes trade valuable resources with one another to promote their own growth. These intercellular processes occur prevalently in natural microbial communities and are at the heart of a complex chain of cooperative behaviors that exist throughout the biosphere [1–5]. Microbes are known to engage in the exchange of a variety of metabolites including essential amino acids, sugars, fatty acids and cofactors [6–8] that drive the dynamics, stability and evolution of microbial communities, which are only beginning to be fully elucidated.

Past efforts to study microbial metabolic exchange have led to crucial insights regarding the dynamics [9–13], energetics [14, 15], and evolutionary origin [16–19] of this multifactorial process [20]. Several experimental models of microbial metabolic interactions have been developed in proteobacteria, yeast, and archaea [10, 11, 21]. Recent theoretical efforts using constraint-based models [22] such as Flux-balance analysis (FBA) and dynamic FBAs have extended single-cell metabolic models to community-level metabolic reconstructions to investigate population level properties [23, 24] and dynamic processes [25, 26]. These approaches have yielded fruitful insights that deepen our understanding of microbial communities and inform better predictions of their dynamics [27, 28]. More recently, the idea of biological markets [29–31] has been suggested as a way to study microbial interactions [32].

**Fig 1:**
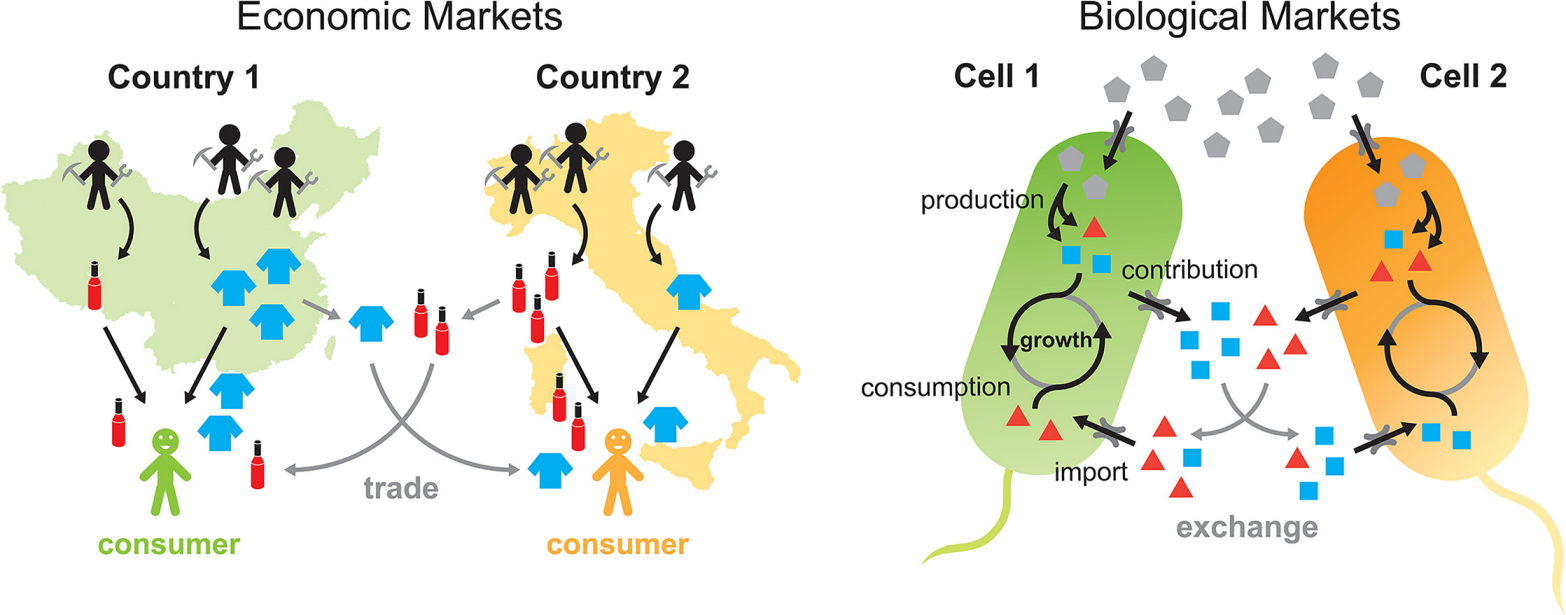
Similarities between economic markets and biological markets. **(a)** Market trade between countries where labor is used to produce goods. A unit of labor can produce 1 wine or 3 cloth in *Country 1* and 3 wine or 1 cloth in *Country 2*. Both countries benefit through trade by having *Country 1* trade cloth to *Country 2* for wine. Demand drives production and consumers purchase goods to maximize their utility. **(b)** Metabolic exchange between microbial cells. Microbes convert an input resource (grey symbol) into metabolites needed for growth (colored squares/triangles). For each unit of input, *Cell 1* can produce either 2 square metabolites or 1 triangle metabolite, while *Cell 2* can produce either 1 square metabolite or 2 triangle metabolites. *Cell 1* exports its squares and *Cell 2* exports its triangles into the environment, where metabolites mix and are imported back into cells. Metabolites are then used for growth as cells tune their production levels to maximize individual growth rates.

Conceptually, a microbial population exchanging metabolites is similar to an economic market (Fig 1). Microbes possess the ability to convert various resources (e.g. sugars, metabolites) into other forms (e.g. amino acids) that are used for growth. Cells have influx and efflux pumps to transport metabolites between their intracellular compartment and their local extracellular environment. In natural habitats, cells are surrounded by thousands of different metabolites generated by their neighbors who may possess different physiologies and complementary metabolic capabilities. These environments are ripe with opportunities to engage in mutually beneficial metabolic trades. Thus, microbes can trade resources with one another to promote their own growth just as countries trade with their neighbors to increase their material wellbeing.

Economists have been studying trade and markets for more than a century. The research culminated in the 1950’s with general equilibrium theory (GET), a mathematical framework that attempts to explain the behavior of markets [33]. GET models a centralized market in which consumers sell their endowed goods (e.g. labor) in order to purchase desired goods, and firms transform input goods to output goods in order to obtain profit. An equilibrium exists when, consumption, production, and exchange are individually optimal for each agent, and there is no oversupply or shortage of goods at the prevailing price. Because of the striking similarities between economic markets and microbial metabolic exchange, GET could be adapted to understand biological markets, providing a new and powerful framework for microbial ecology.

Here, we present biotic general equilibrium theory (BGET), a mathematical framework that explicitly integrates economic concepts to study metabolic exchange in microbial communities. For each member of a community, BGET takes its metabolic capabilities, resource requirements for growth, and rate of resource exchange as input parameters and computes the consumption, production, and exchange of resources. Based on these variables, the model is able to determine the growth rate of each member of the community. BGET provides a theory of microbial trade that predicts the mutualistic benefits of resource exchange from first principles. The model is scalable to an arbitrary number of species and metabolic interactions. We applied this model to analyze two bacteria engaging in metabolic exchange, which yielded interesting and important properties of a simple microbial community. Furthermore, we used a synthetic *Escherichia coli* co-culture engaged in auxotrophic exchange of essential amino acids to experimentally confirm a key implication of the model. This BGET framework provides a new perspective to study key questions in microbial ecology.

## Model: Biotic General Equilibrium Theory (BGET)

**Fig 2:**
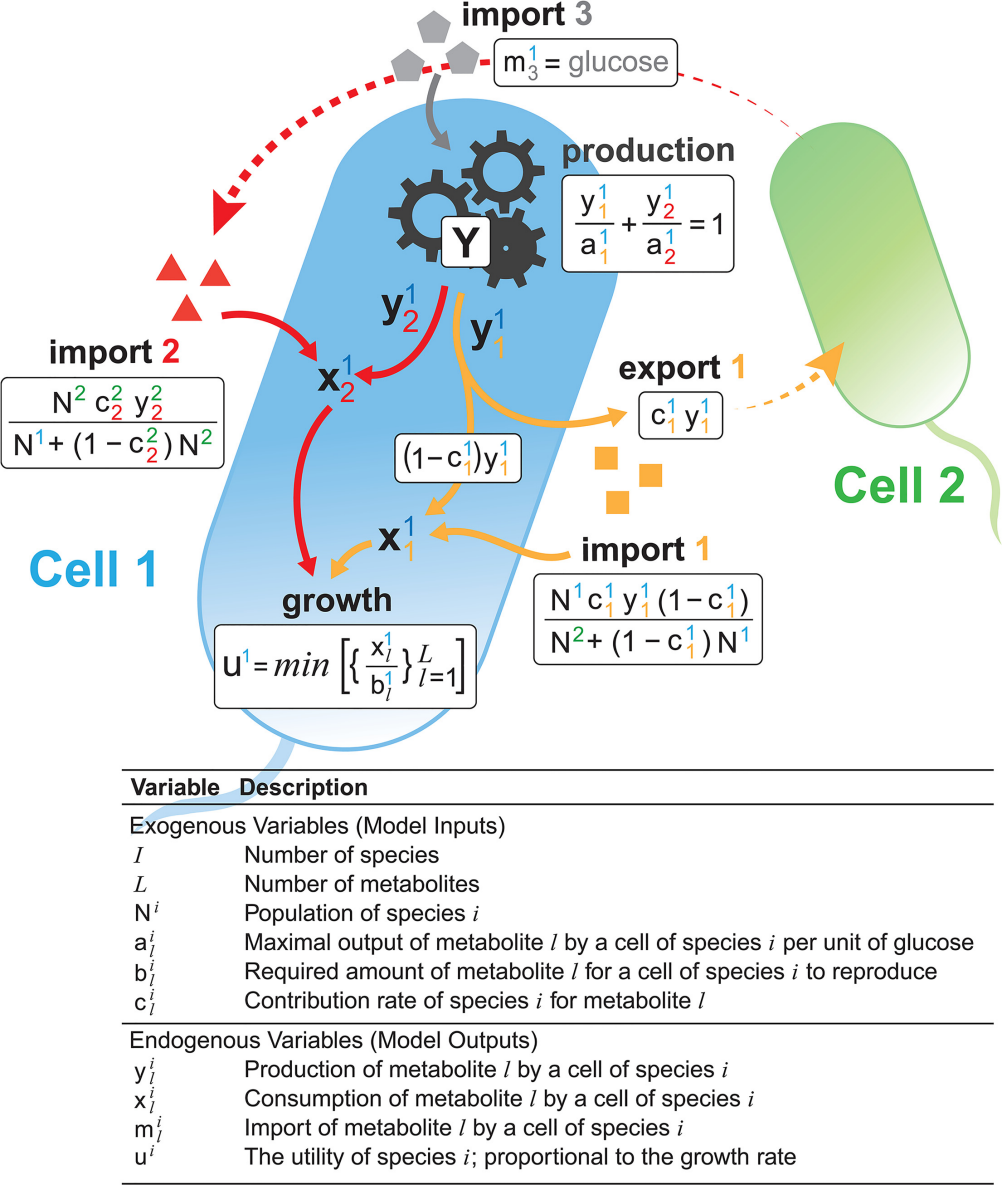
Biotic Equilibrium in the 2x2 Model. Cell 1 takes glucose $m_{3}^{1}$ and uses it to produce the orange *metabolite-1* $y_{1}^{1}$, and the red *metabolite-2* $y_{2}^{1}$, A fraction $c_{1}^{1}$, of *metabolite-1* production is exported and a fraction of the export is then re-imported. The cell also imports red *metabolite-2* from cell 2. The imports plus the production for each metabolite equals the consumption of each metabolite $x_{1}^{1}$ and $x_{2}^{1}$, facilitating growth *u* ^1^.

Consider a community of microbes in a well-mixed environment, which contains nutrients that each microbe can use to grow and produce various metabolites. Some of these metabolites are released back into the environment where they are taken up by other microbes for utilization. For simplicity, let us consider a community containing two distinct species, each able to convert one primary resource abundantly available in the medium (e.g. sugars) into two additional metabolites (e.g. amino acids) that then can be readily exchanged through membrane transporters. Both species need the two produced metabolites for growth, and both possess the metabolic pathways necessary to generate them. Biotic general equilibrium theory (BGET) takes the growth needs, metabolic capabilities, and intercellular transport rates of each species and determines the production and allocation of metabolites, and instantaneous growth rate of each species (Fig 1). To determine the population dynamics over time, BGET is then iterated over a discrete-time framework. We refer to “variables” as outputs of the model and “parameters” as inputs. In the main text of this paper, we describe the basic intuition of the model using a 2-member community as an example and focus on stable steady-state population dynamics. A formalized description of the 2-member model is presented in the *Materials and Methods* section. A list of parameters and variables and a diagram that depicts their relations is presented in Fig 2. The general BGET model that allows for an arbitrary collection of species, metabolites, and metabolic interactions is also detailed in the *Materials and Methods* section.

### Production and Consumption

We will use the superscript *i* to indicate the microbial species and the subscript *l* to indicate the metabolite. Each cell possesses metabolic pathways that allow it to convert input metabolites to output metabolites. Here, we consider that the input resource (e.g. sugar) is not limiting, but uptake from the environment per unit of time is fixed to be one. There are two possible output metabolites. The production of metabolite *l* by a cell of species *i* is given by the variable $y_{l}^{i}\geq 0$. This is the amount of metabolite that the cell produces in a single period of time. For each species, the maximum production is constrained by the equation $y_{1}^{i}/a_{1}^{i}+y_{2}^{i}/a_{2}^{i}=1$. The productivity parameters $a_{1}^{i}>0$ and $a_{2}^{i}>0$ here represent the amount of output possible per unit of input (e.g. sugar). The number 1 on the right-hand side of the equation represents the uptake of input resource. This means that if the cell devotes all of its energy to the production of *metabolite-1*, it can produce up to $a_{1}^{i}$ units and if the cell devotes all of its energy to the production of *metabolite-2*, it can produce up to $a_{2}^{i}$ units.

Each species *i* requires various amounts of metabolite *l* to grow, which is captured by the resource requirement parameter $b_{l}^{i}\geq 0$. As such, metabolite consumption fuels cellular growth. The consumption of metabolite *l* by species *i* is given by the variable $x_{l}^{i}\geq 0$. In order to maximize its growth rate, each cell will adjust its production of metabolites. In GET, people are assumed to maximize a utility function, which represents a person’s preference ordering of goods, measured in terms of utils. For example, a bundle of 2 apples, 1 car, and 1 house may have a value of 10 utils, and a bundle of 3 apples, 2 cars, and 0 houses may have a value of 7 utils. In GET, a person chooses the bundle with the highest value to maximize his or her utility. Similarly in BGET, the resource requirements for growth imply an ordering of metabolite bundles favoring those that yield more growth, and cells adjust their production to consume the metabolite bundle that maximizes their growth. Therefore in the biological case, growth is equivalent to utility. We let *u* ^*i*^(*x* ^*i*^) represent the utility function of a cell of species *i*. Since a cell cannot substitute one type of metabolite for another, a cell maximizes its utility by maximizing its consumption of its limiting metabolite. In economics, this is analogous to Leontief preferences [33]. A notable difference between the economic and biological contexts is that in economics utility is a purely theoretical and unmeasurable construct. It is a way to represent preference. If a consumption bundle *x* is preferred to *x*', then *x* is said to generate more utility than *x*', but utility itself is never directly observed. Barring, perhaps mind-reading technology, utility is not directly observed. In the biological context however, utility has a physical manifestation–the cell’s growth rate. In this way, BGET applied to microbes is actually observationally easier to analyze than GET applied to human markets.

### Exchange

Cells may exchange metabolites for mutual benefit. In our model, each species *i* has a contribution parameter $c_{l}^{i}\geq 0$, which is the fraction of the produced metabolite *l* that is exported into the shared environment. Without loss of generality, we explore the case when *species-1* exports only *metabolite-1* and *species-2* exports only *metabolite-2* (see *the* Materials and Methods *section* for the general model). Import of metabolite *l* for species *i* is given by $m_{l}^{i}\geq 0$. We assume that the maximum amount of metabolite *l* that species *i* can import per period is given by ${\bar{m}}_{l}^{i}\geq 0$. This import cap accounts for the physical limits of metabolite import, which prevents the growth rate from reaching non-physiological values. The last component of the model is an allocation rule that relates how cells import metabolites. In human markets, prices equilibrate the supply and demand for a given good. In the biotic market, resources are shuttled in and out of the cell via membrane transporters. The basic idea behind the exchange process represented in the model is that cells with equal contribution rates of metabolite *l* will import equal amounts as long as they need the metabolite. A cell does not import metabolites in excess of its needs. Since a cell of a species with a strictly positive contribution parameter will re-export metabolites that were imported, its import rate must be reduced as illustrated by the following example. Suppose *species-1* contributes *metabolite-1* at $c_{1}^{1}=0.7$, whereas *species-2* does not contribute *metabolite-1* $\left(c_{1}^{2}=0\right)$. Furthermore, there is a large supply of *metabolite-1* in the environment, and each cell does not import more than 10 units of *metabolite-1* per period, ${\bar{m}}_{1}^{i}=10$. A cell of each species will initially import 10 units of *metabolite-1*. However, since a cell of *species-1* re-exports 7 of these 10 units back into the environment, its net import will be only 3. Thus a cell of *species-1* imports only 3 units for every 10 units that a cell of *species-2* imports. Assuming cells from both species require metabolite *l* for growth and have not reached their maximum import ${\bar{m}}_{1}^{i}$, then the ratio of metabolite import between a cell of each species is $\left(1-c_{l}^{i})/(1-c_{l}^{j}\right)$. In general, rates of metabolic exchange can change depending on how the membrane transport machineries are regulated. We first take the contribution rate for a given species as fixed. Later, we explore the impact of varying the contribution rate on the cell population.

### Equilibrium

The driving mechanism underlying the BGET model is that a cell adjusts its production levels to maximize its utility function given the available metabolites, thereby maximizing its growth rate. Given the production capability, contribution rates, and imports, the cell uses its metabolic pathways in a manner that maximizes utility by producing the appropriate metabolites. Since the cell’s utility function, import-export processes, and constraints can all be written as linear equations and expressions, the solution to the model–biotic equilibrium–can be solved using standard linear programming methods [34]. While the solution to the model is difficult to represent as an explicit equation, linear programming enables us to efficiently compute numerical solutions.

**Fig 3:**
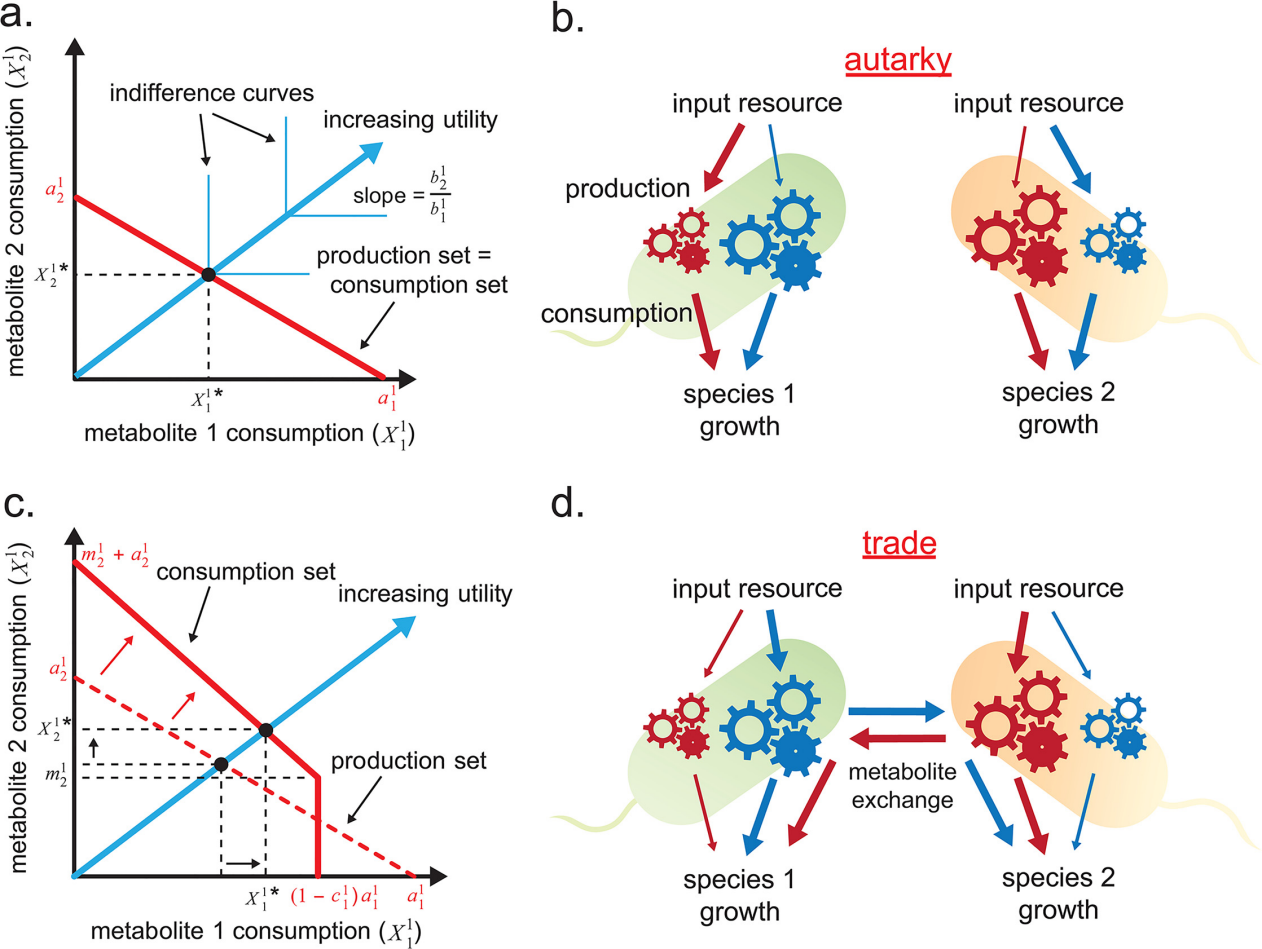
Production and consumption of a *species-1* cell in a co-culture. **(a)** Population equilibrium under autarky (no trade). The blue arrow represents the direction of increasing utility. Consumption of the metabolites in the ratio $b_{2}^{1}/b_{1}^{1}$ lead to growth. The *indifference curves* are contour lines of the utility function: any two consumption vectors on the same indifference curve lead to the same growth rate. The solid red line represents the possible production vectors of *metabolite-1* and *metabolite-2*. The intersection of the blue arrow with the consumption-set line is the equilibrium consumption vector for which utility is maximized. When there is no trade the consumption and production sets are equivalent. The cell can only consume what it produces. **(b)** Schematic of cells under autarky. Arrows represent the flow of resources during production and consumption of red and blue metabolites. The arrow width illustrates the utilization of each pathway. The gear size indicates pathway productivity. Cells need to allocate more input resources to produce metabolites for which it has lower productivity, given that utilization requirements are about equal. **(c)** Population equilibrium under trade where the consumption set is expanded (solid red line). The consumption set line shifts upward by the amount *species-1* imports from *species-2*, $m_{2}^{1}$. The increased magnitude of the slope of the consumption set line indicates that a fraction of *metabolite-1* produced is exported to *species-2*. The slope is given by $-(a_{2}^{1}/(1-c_{1}^{1})a_{1}^{1})y_{1}^{1}$. The new equilibrium consumption vector and utility level is greater under trade than autarky implying higher growth rates. **(d)** Schematic of trading cells. A cell can allocate most of its input resources to produce metabolites for which it has high productivity when exchange occurs.

The intuition for equilibrium can be expressed graphically (Fig 3). Under autarky (defined as no trade), the set of metabolites that could be produced is the same as the set that can be consumed. The cell adjusts production to obtain the maximum possible utility depicted by the intersection of the blue arrow and the red line (Fig 3A). If a cell of *species-1* needs an equal amount of *metabolite-1* and *metabolite-2* for growth, and is more productive at generating metabolite 1, then the cell will devote relatively more input resources (e.g. glucose) to producing metabolite 2 (red arrows of Fig 3B). With trade, however, a cell of *species-1* can receive *metabolite-2* from *species-2*. Trade shifts the consumption set upwards by the amount of import, and it increases the magnitude of the slope of the consumption in proportion to the amount that the cell exports (Fig 3C). Thus, trade can increase the cell’s total consumption and hence increase its growth in a mutually beneficial fashion (Fig 3D).

### Dynamics

**Fig 4:**
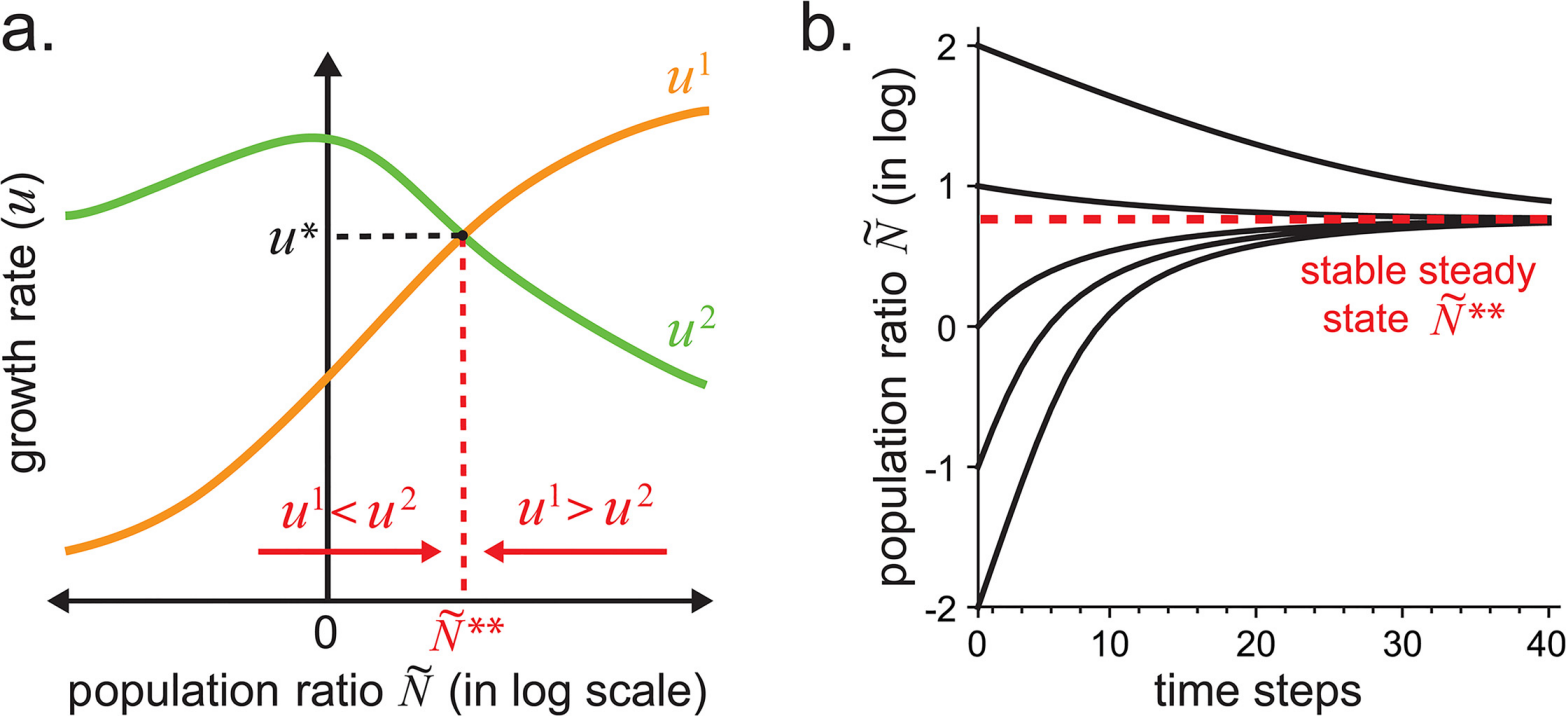
Population dynamics. **(a)** Growth rate as a function of the log population ratio. When *u* ^1^ < *u* ^2^ the population ratio increases, and when *u* ^2^ < *u* ^1^ the population ratio decreases. When *u* ^1^ = *u* ^2^ the population ratio does not change. The steady state ${\tilde{N}}^{**}$ is stable since small perturbations in the population ratio lead the population ratio back to ${\tilde{N}}^{**}$. **(b)** Log population ratio over time after a perturbation at time step 0. Population converges back to SSS over time.

The biotic equilibrium specifies the growth rate of both species at a single point in time. To capture the population dynamics over time, the population level is updated according to the equation, *N* ^*i*^(*t* + 1) = (1 + *u* ^*i*^(*x* ^*i*^(*t*)))*N* ^*i*^(*t*), where *x* ^*i*^(*t*) denotes a cell of species *i*’s consumption at time *t*, and *N* ^*i*^(*t*) denotes the population level of species *i* at time *t*. While the model captures the population dynamics, however complex, we will focus on analyzing the steady-state outcomes in this paper. We define $\tilde{N}=N^{2}/N^{1}$ as the population ratio between species. As the population ratios change, the biotic equilibrium also changes. The population ratio is said to be in steady state, denoted by ${\tilde{N}}^{*}$, when the growth rates of both species are equal. The steady state is stable, denoted by ${\tilde{N}}^{**}$, if small perturbations in the population ratio lead the population to converge back to steady state. The formal definition of a stable steady state (SSS) is provided in Methods Section. Since many microbial communities exhibit SSS behavior, we focus on understanding these SSS processes. For a 2-member community, the steady state population ratio ${\tilde{N}}^{**}$ is stable when small decreases in the population ratio cause growth rate differences *u* ^2^ > *u* ^1^ and small increases cause *u* ^1^ > *u* ^2^, leading to convergence back to *u* ^1^ = *u* ^2^ = u* (Fig 4A). Sometimes, environmental stresses (e.g. exposure to antibiotics) can perturb the growth of one or more members of the community, leading to population disequilibria out of its steady state. The BGET model can thus be used to study the population dynamics as the community relaxes back to its stable steady state (Fig 4B). Various shock analyses can be applied to study the rate of reversion back to stable steady state after environmental perturbations.

## Results

### Comparative Advantage is Necessary for Mutualistic Exchange

In 1817, David Ricardo first observed the law of comparative advantage [35]. If productivity is defined as the amount of output of a particular good per unit of input, then the law of comparative advantage states that there are mutual gains from trade as long as two trading agents produce goods at different productivity ratios. This is surprising because mutual gains from trade exist even when one agent has an absolute advantage in producing all goods. Economists view comparative advantage as one of the fundamental principles that drive trade in human economies. The same principle applies in the context of our BGET model. Under autarky (no trade), each species has to devote a large fraction of its input resources to produce the metabolite for which it has low productivity (Fig 3B, red arrows for *species-1* and blue for *species-2*). With trade, each species can obtain those low-productivity metabolites from its trading partner while diverting more resources to produce high-productivity metabolites (Fig 3D, blue arrows for *species-1* and red for *species-2*). Thus, trade allows species to specialize in metabolites they are better at producing, thereby benefiting the entire population. For example, we can say that *species-1* has a comparative advantage in *metabolite-1* and *species-2* has a comparative advantage in *metabolite-2* if the productivity parameters $a_{1}^{1}/a_{2}^{1}>a_{1}^{2}/a_{2}^{2}$. When $a_{1}^{1}/a_{2}^{1}=a_{1}^{2}/a_{2}^{2}$, there is no comparative advantage between the two species. Comparative advantage is a necessary condition for mutualistic exchange. A simple demonstration is presented in the *Supporting Information* S1 Appendix *–Comparative Advantage is Necessary for Mutualistic Exchange*. Because different species in a microbial consortium often possess different metabolic capabilities, they may often have different productivities for different metabolites that are being exchanged. Thus, the law of comparative advantage is likely an important driver of microbial population dynamics.

### Comparative Advantage Is Necessary for Stability

**Fig 5:**
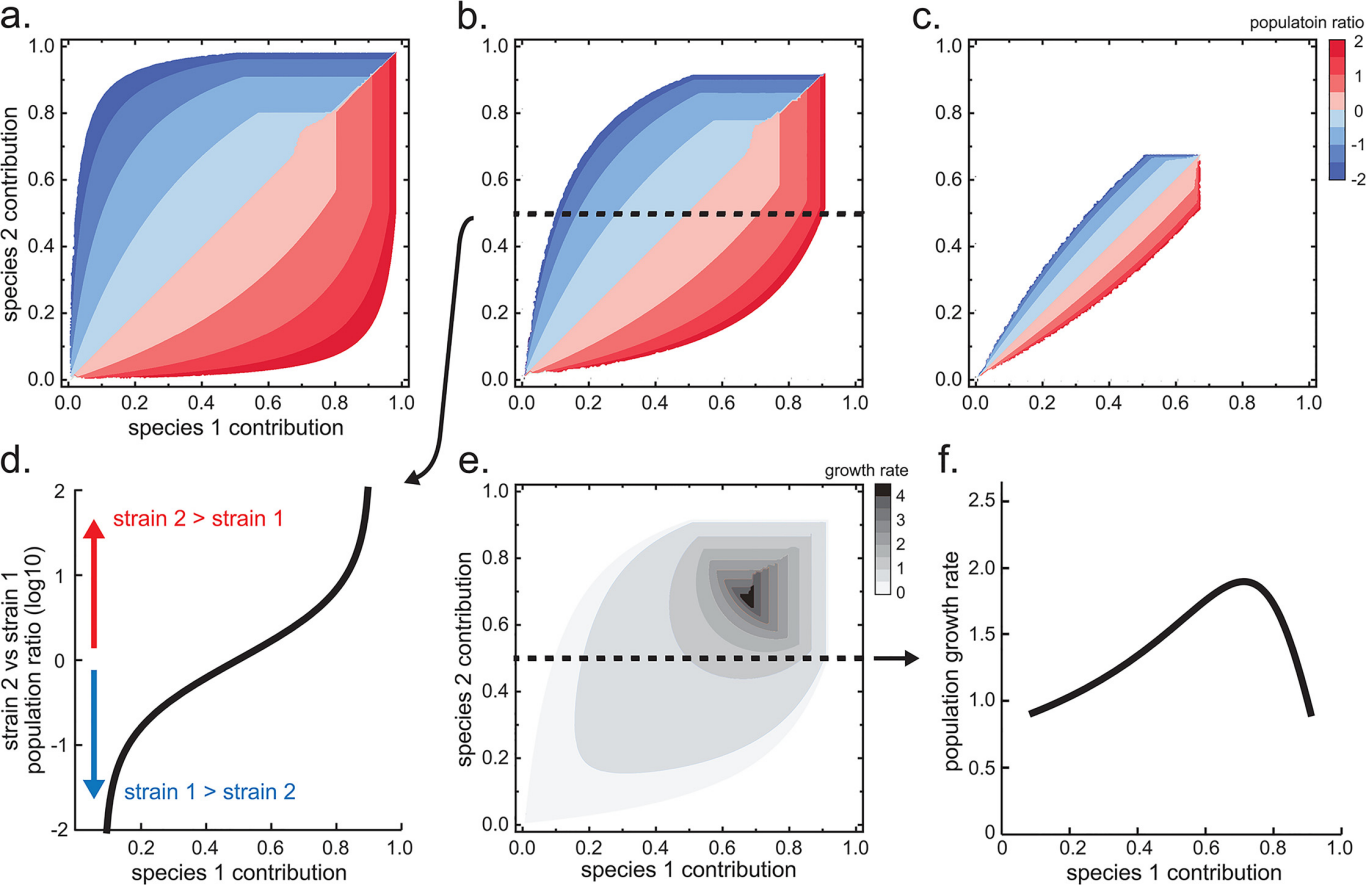
(a)-(c) Stable contribution-space of three populations with decreasing comparative advantage. Horizontal and vertical axes are contribution rates of *species-1* and *species-2* respectively. Productivity parameters are $a_{i}^{i}=100$, $a_{j\neq i}^{i}=1$, ${\bar{m}}_{l}^{i}=100$ for **(a)**, $a_{i}^{i}=10$, $a_{j\neq i}^{i}=1$, ${\bar{m}}_{l}^{i}=10$ for **(b)**, and $a_{i}^{i}=2$, $a_{j\neq i}^{i}=1$, ${\bar{m}}_{l}^{i}=1.3$ for **(c)**. Color contours are the population ratio of *species-2* versus *species-1* in the form of *log*(*N* ^2^ / *N* ^1^). The stable contribution-space shrinks as comparative advantage decreases. **(d)** Dotted line cross-section from plot (b) showing population ratio as a function of *species-1* contribution $c_{1}^{1}$ for a fixed *species-2* contribution of $c_{2}^{2}=0.5$. **(e)** Populations growth rate of plot (b) with darker contours representing increased growth rate. **(f)** Dotted line cross-section from plot (e) showing growth rate as a function of *species-1* contribution $c_{1}^{1}$ for a fixed *species-2* contribution of $c_{2}^{2}=0.5$.

It remains an open question whether comparative advantage is necessary for two populations to live together in SSS. We consider a community of two trading species where the productivity ratios of the two species are reciprocals of each other, that is $a_{1}^{1}/a_{2}^{1}=a_{1}^{2}/a_{2}^{2}$. This means that, for example, a *species-1* cell may produce four molecules of *metabolite-1* and two of *metabolite-2* for every glucose molecule consumed, and a *species-2* cell may produce two of *metabolite-1* and four of *metabolite-2* for every glucose molecule consumed. By definition, as this productivity ratio $a_{1}^{1}/a_{2}^{1}$ increases so does the comparative advantage. For simplicity, we assume that the growth requirements for each metabolite for both species is one (i.e. $b_{l}^{i}=1$). Thus for populations with different magnitudes of comparative advantage, there exists different sets of SSS (Fig 5A–5C). An increase in the metabolite contribution rate by a species will move the SSS along the surface where the contours indicate the population ratio *log*(*N* ^2^ / *N* ^1^). Here, we refer to the contribution-space that produces SSS as the “stable contribution-space”. When there is no comparative advantage (i.e. $a_{1}^{1}/a_{2}^{1}=a_{1}^{2}/a_{2}^{2}$), the stable contribution-space is empty. An increase in comparative advantage leads to a larger stable contribution-space (Fig 5A–5C). The analysis indicates that for this setup, comparative advantage is necessary for SSS. Further discussions on the shape and intuition regarding the stable contribution-space can be found in the *Supporting Information* S1 Appendix.

### Natural Selection for Auxotrophy

Auxotrophs, or cells unable to produce essential metabolites, are prevalently found in nature [13, 36]. Based on BGET, an autotroph, a cell able to produce all essential metabolites, will expend resources on generating metabolites that it does not trade and for which it is comparatively less productive than its trading partner. An auxotroph is missing this metabolic pathway and instead expends its resources on producing its exporting metabolite. Thus auxotrophy precludes cells from allocating resources in a communally inefficient manner, thereby potentially generating a higher growth rate for the whole population. The loss of function operates as a commitment that prevents the species from doing what is individually optimal, but suboptimal collectively. Thus with strong group-selection pressures, BGET suggests that auxotrophy may arise as an adaptation.

We illustrate this concept through a simple numerical example. Let the productivity of autotrophic *species-1* be $a_{1}^{1}=100$, $a_{2}^{1}=1$, and autotrophic *species-2* be $a_{1}^{2}=1$, $a_{2}^{2}=100$ with ${\bar{m}}_{1}^{i}=40$ for both species. Here, there is a significant comparative advantage between the autotrophs ($a_{1}^{1}/a_{2}^{1}>a_{1}^{2}/a_{2}^{2}$), where *species-1* can make *metabolite-1* far better than *metabolite-2*, and *species-2* can make *metabolite-2* far better than *metabolite-1*. There is the potential for large gains through trade in the autotrophic community. Let us now assume that *species-1* becomes auxotrophic for *metabolite-1* and *species-2* auxotrophic for *metabolite-2* (i.e. $a_{1}^{1}=100$, $a_{2}^{1}=0$, $a_{1}^{2}=0$, $a_{2}^{2}=100$). Interestingly, the BGET model suggests that the maximum SSS growth rate of this auxotrophic population will be ~30% higher than that of the autotrophic population. The competitive advantage of auxotrophic cells over autotrophic cells was recently shown in synthetic *E*. *coli* co-culture populations [37], thus highlighting an opportunity to engineering such phenotypes for industrial applications of multi-species microbial communities [38].

This is distinct from other theories that explain the existence of auxotrophy. It is believed that auxotrophs can arise naturally by gene loss during evolution through “genome streamlining”, and can often outcompete cells that are autotrophic in nutritionally rich environments [36]. Our mechanism also differs from the “Black Queen Hypothesis” in which auxotrophy is selectively favored at the individual level to reduce a costly and leaky function (e.g. biosynthesis of amino acids) [39].

### Growth-Relative-Abundance Tradeoff

The BGET model predicts that there is an important tradeoff between the population growth rate and the relative species abundance. In some environments selective forces may cause species to maximize relative abundance. In environments where group selection plays an important role, species may be pressured to maximize their group’s growth rate. The model makes clear that there is a trade-off between these two strategies. As a species increases its contribution, its relative SSS abundance in the population decreases (Fig 5D). However, the population growth rate is maximal at an intermediate contribution level (horizontal line, Fig 5D). Beyond this point, the growth rate will decrease and species that continue to increase in contribution will further decrease in relative abundance. Thus, under- and over-contribution both lead to sub-maximal population growth, thereby highlighting a potential evolutionary driver for the development of optimal rates of metabolic exchange. Since under-contribution leads to higher relative abundance in the community, there is a tradeoff between relative population abundance and growth. If a species reduces its contribution then its relative abundance will increase, but at the expense of a lower overall population growth rate. In other words, because the growth-maximizing contribution rate and the relative-abundance maximizing rate do not coincide, there will always be a tradeoff between these two. In nature, however, different environments may promote different strategies along this growth-relative-abundance spectrum. Certain opportunities selectively favor maximal growth rate (e.g. rapid colonization of a new niche) whereas others may favor population abundance (e.g. quorum sensing in biofilms). Thus, the variation in species abundances observed in nature may be governed in part by this growth-relative-abundance tradeoff.

**Fig 6:**
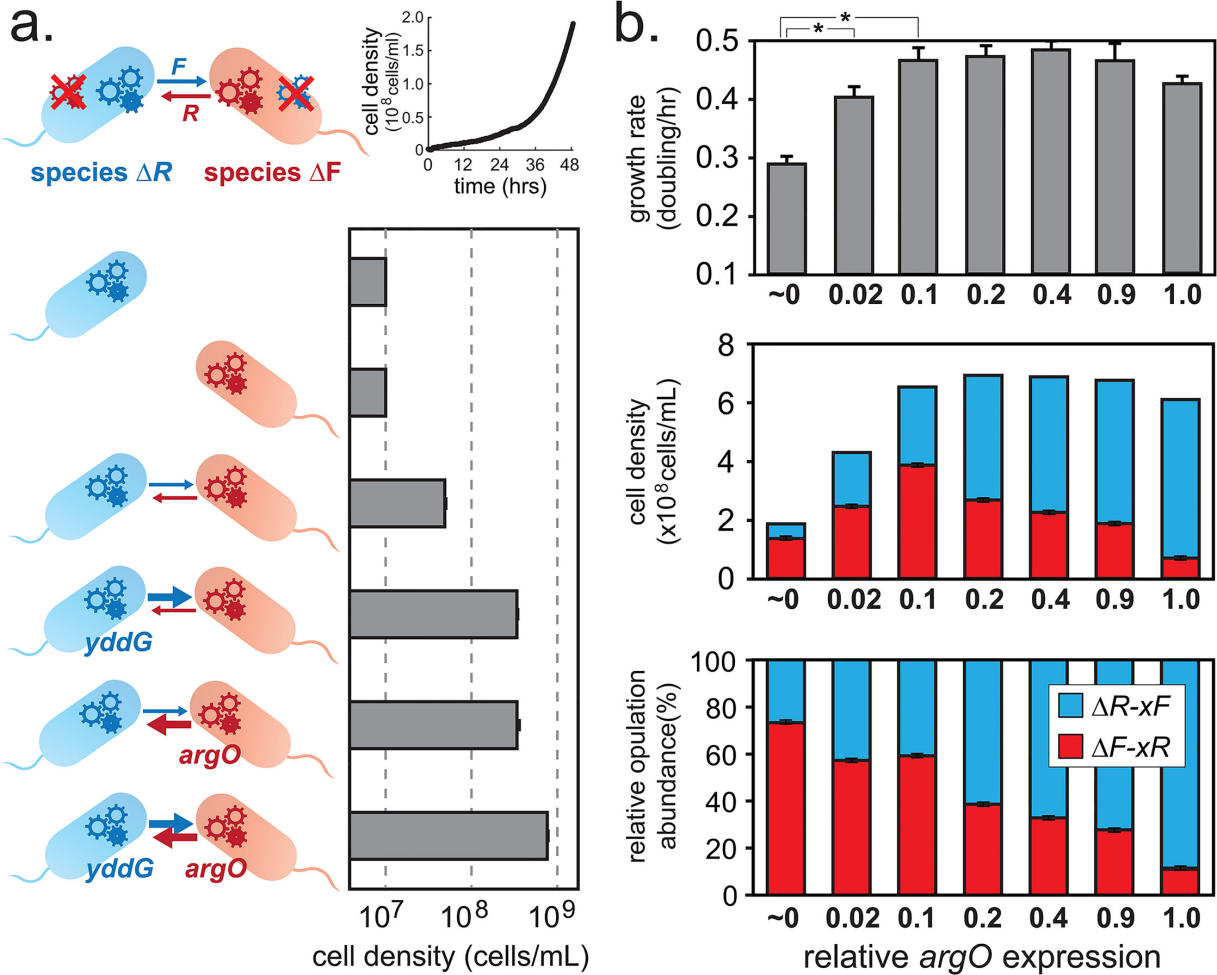
Experimental measurement of growth-abundance tradeoffs. All data are averages from experiments in biological replicates (n = 3). **(a)** Auxotrophic *E*. *coli* strains *∆R* and *∆F* grow by exchange of metabolites arginine ***R*** and phenylalanine ***F*** in a co-culture (black growth curve). Bar graph shows 24-hr population density of co-culture variants with ***R*** and ***F*** amino acid exporters *argO* and *yddG* respectively. **(b)** Growth rate, 24-hr cell density, and population ratio (top, middle, bottom panels) of co-cultures of *∆R-xF* and *∆F-xR* where *argO* expression is increased on a relative scale of 0 to 1. Asterisks highlight statistical significance (p<0.001 by t-test). Error bars indicate standard deviations of data acquired in biological replicates (n = 3).

To further explore the growth-relative-abundance tradeoff predicted by BGET, we developed a simple experimental bacterial consortium using two auxotrophic *E*. *coli* strains that engage in metabolic exchange. Each strain is unable to synthesize an essential amino acid (phenylalanine ***F*** or arginine ***R***) and thus cannot grow individually in minimal media (M9-glucose). However, both auxotrophic strains (designated *ΔF* or *ΔR*), when combined, are able to grow by intercellular metabolic exchange of ***F*** and ***R*** (Fig 6A). To modulate the degree of metabolic exchange, we built *ΔF* and *ΔR* variants that increase their amino acid contributions by activating specific amino acid efflux pumps. The *ΔF-xR* strain possesses increased ***R*** export through a tunable *argO* gene [40] while the *ΔR-xF* strain possesses increased ***F*** export through the *yddG* gene [41]. Co-cultures of these variants showed significant increases in growth rate when compared to their ancestral strains (*ΔF* and *ΔR*) (Fig 6A). We then systematically generated *ΔF-xR* variants with increased ***R*** contribution by tuning *argO* gene expression (see Methods). While using a *ΔR-xF* strain with a constant ***F*** contribution, we separately co-cultured this *ΔR-xF* with each of the *ΔF-xR* variants and measured the population growth rate and relative abundance of each species. We find that increasing ***R*** contribution by *ΔF-xR* variants significantly increased the overall population growth rate up to a peak before declining, but always reduced the relative population abundance of the *ΔF-xR* variant (Fig 6B). We therefore confirmed the same type of grow-relative-abundance tradeoffs in our experimental system as what was predicted by the BGET model. At low contribution rates, any increase in contribution leads to an initial increased population growth, but higher contribution rates always lead to a lower relative abundance and a lower overall growth rate. Interestingly, we also find an initial tradeoff between relative and absolute abundance. For example, even though *ΔF-xR* relative abundance at contribution level 0.1 is lower than at contribution level ~0 (relative abundance of 59% vs. 73%), its absolute abundance is actually higher (3.8x10^8^ vs. 1.4x10^8^ cells after 24 hours).

## Discussion

The BGET framework leverages fundamental insights from economics to help elucidate important properties of microbial metabolic exchange and population dynamics. Our model of microbial trade yields several insights, including the notions that comparative advantage enhances stable population growth, trading auxotrophs can be evolutionary favored, and growth-relative-abundance tradeoffs manifest in exchanging populations, which we also experimentally confirmed.

The model provides a framework to think about important evolutionary questions. For instance, where do productivity differences come from? In economic models, productivity differences from countries are often believed to come from differences in resources, differences in the skills of the populace, and differences in technologies. Why might different species have differences in productivity ratios (i.e. comparative advantage)? Natural selection may optimize species' productions in specific environments. Species from different environment may come together as a consortium already endowed with productivity differences. Alternatively, if the adaptations that make a cell very productive at producing one metabolite also make the cell less productive at producing another metabolite, then natural selection may cause species with identical productivities in a consortium to divergently evolve. Such consortia that specialize and trade will outperform consortia of non-trading generalists. Additionally, the model shows that trade leads to mutual benefits, but it is clear that a mutant free-rider species with a zero contribution rate, but otherwise identical to a trading species in the consortium can invade. How can trading consortia exclude such free-riders? While this question is beyond the scope of this paper, the BGET model provides a framework to compare the growth of consortia with and without trade allowing for analysis of multi-level selection. Future extensions of the model may include mechanisms, such as positive feedback loops (a cell exports only if it receives imports), that may increase the resistance of consortia to invasion.

BGET bears some similarities and differences with other models of microbial communities. A recent model by Enyeart et al. provides a formal mathematical treatment of microbial communities, explicitly using the economic concept of comparative advantage [42]. However, their model is a system of ordinary differential equations in which a species produce an output that collectively benefits the community as a whole (e.g. proteins that enable antibiotic resistance). In contrast, BGET models the production and exchange of metabolites between cells as opposed to the production of public goods that directly benefit the whole community. Conceptually, BGET may also appear similar to FBA models since they both employ linear programming methods to find optimal solutions for cellular metabolism. However, BGET differs from FBA primarily in that it uses a higher level of abstraction by focusing on the inputs and outputs of metabolism. We see two advantages to this approach. First, it clearly delineates economic objects allowing for clear application of economic principles. This is analogous to the way high-level computer programming languages allow for easier manipulation of higher-level structures than machine language or assembly language, although in principle a given algorithm could be represented isomorphically in either language. Second, FBA often requires whole-cell metabolic reconstructions, which are often incomplete or unavailable. By focusing on the inputs, outputs, and requirements for growth, BGET simplifies this problem allowing for predictions in absentia of complete data. Here, we do not account for stoichiometric parameters, which may limit the precision of the model predictions in certain instances. [26]. Further improvements could enhance BGET by including additional stoichiometric constraints.

Beyond what is described here, other economic concepts may further improve the understanding of microbial ecology, such as vertical integration. A species that chooses to produce all of its own resources instead of outsourcing these activities to other species in the community is performing the biological equivalent of vertical integration. In economics markets, these vertically integrated firms produce all the units in their supply chain. Understanding the key drivers that lead to the formation of vertically integrated species will likely yield new insights not only for microbial ecology but also for validating economic principles using well-controlled synthetic microbial systems as shown here. Furthermore, application of dynamic analysis to history- or density-dependent non-steady-state growth could capture other interesting properties of the population dynamics. Spatial heterogeneity, an important feature of microbial biofilms and structured environments [43], can be integrated to further enhance the BGET framework. These and other extensions, both theoretical and experimental, could lead to new insights for the economics of resource exchange in biological communities at many spatiotemporal and organismic scales.

## Materials and Methods

We introduce the model with the simple 2x2 case in which there are two species and two output metabolites and then present the general model.

### Simple 2x2 Model

Let *i* ∈ {1,2} indicate the species, and let *l* ∈ {1,2,3} indicate the metabolite. The production set *Y* ^*i*^ is the set of all production vectors that are possible for an organism of species *i*. The vector *y* ^*i*^ ∈ *Y* ^*i*^ gives the net outputs of the production process. We will set metabolite *l* = 3 to represent glucose. The production set is

$$Y^{i}=\{y^{i}|y_{1}^{i},y_{2}^{i}\geq 0,y_{3}^{i}\leq 0,y_{1}^{i}/a_{1}^{i}+y_{2}^{i}/a_{2}^{i}\leq -y_{3}^{i}\}.$$

We set $b_{3}^{i}=0$, which means that cells do not use glucose directly to reproduce, the glucose is used only as an input to produce *metabolite-1* and *metabolite-2*. Assume that the maximal import of glucose for both species is one, ${\bar{m}}_{3}^{i}=1$. This implies that $y_{3}^{i}=-1$. The negative value indicates that the net production of glucose is negative, or in other words that glucose is an input in production and not an output. This term binds the growth rates of the species. A cell at maximal output has production given by $y_{1}^{i}/a_{1}^{i}+y_{2}^{i}/a_{2}^{i}=1$ as presented in the main text. The objective function of a cell of species *i* is $u^{i}(x^{i})=\min({\left\{x_{l}^{i}/b_{l}^{i}\right\}}_{l\in \{1,2\}})$. To be clear, all values in this model represent levels and not concentrations.

The *biased-access allocation* rule determines imports as a function of existing exports. *Access* is an upper bound on the amount of a metabolite that a cell can import. A cell will not import its full access of a metabolite if the metabolite is not the limiting factor. Otherwise the cell imports its full access. A cell may re-export some of its import and this is a function of its contribution rate $c_{l}^{i}$. When cells are importing their full access a cell of species *i* can import *l relative* to a cell of species *j* is assumed to be $\left(1-c_{l}^{i})/(1-c_{l}^{j}\right)$. This captures the fact that higher contribution rates make importing harder since it goes against active export. One can think of $c_{l}^{i}$ as the fraction of metabolite *l* in cell *i* that is actively transported out of the cell in a unit of time. Consequently each cell of species *i* has initial access to each unit of metabolite *l* equal to $\left(1-c_{l}^{i})/[(1-c_{l}^{i})N^{1}+(1-c_{l}^{2})N^{2}\right]$.

Equilibrium is defined as when every cell maximizes its utility subject to the aforementioned constraints: $max_{x^{i}}u^{i}(x^{i})$ such that, (1) consumption is no more than the sum of production and imports, $x_{l}^{i}\leq m_{l}^{i}+(1-c_{l}^{i})y_{l}^{i}$ if $y_{l}^{i}\geq 0$, and $x_{l}^{i}\leq m_{l}^{i}+y_{l}^{i}$, if $y_{l}^{i}<0$, (2) production is feasible *y* ^*i*^ ∈ *Y* ^*i*^, (3) imports do not exceed the maximum level $m_{l}^{i}\leq {\bar{m}}_{l}^{i}$, and (4) trade satisfies the *biased-access allocation* rule (see below for the formal representation). In summary there are the exogenous parameters $a_{l}^{i}$, $b_{l}^{i}$, $c_{l}^{i}$, *N* ^*i*^ that are taken as inputs into the model. The equilibrium fully determines the values of the endogenous output variables *x* ^*i*^,*y* ^*i*^,*m* ^*i*^ and *u* ^*i*^ of both species.

### General Model

Assume there are *I* species and *L* metabolites. The production set $Y^{i}\subset {\mathbb{R}}^{L}$ is the set of all production vectors that are possible for a cell of species *i*. We assume the production set is nonempty, closed, and convex. The vector *y* ^*i*^ ∈ *Y* ^*i*^ gives the net outputs of the production process. For example, the production vector (0,−2,5,−1) describes the process whereby two units of *metabolite-2* and one unit of *metabolite-4* are converted into five units of *metabolite-3*. For a more in depth treatment of production functions see [33]. The production set is kept quite general. The model allows for any production set that is nonempty, closed, convex, and we impose one additional assumption, that it can be specified with a set of linear constraints. For example, the production set in Simple 2x2 Model represents a system where a single resource is transformed into possibly two metabolites. For another example, $Y^{i}=\{y^{i}|y_{1}^{i},y_{2}^{i}\geq 0,y_{3}^{i},y_{4}^{i}\leq 0,5y_{1}^{i}+7y_{2}^{i}\leq -y_{3}^{i}-y_{4}^{i}\}$, the production set states that *metabolite-3* and *metabolite-4* are used as inputs to produce *metabolite-1* and *metabolite-2* as outputs. A unit of *metabolite-3* or *metabolite-4* can produce 1/5 units of *metabolite-1* or 1/7 units of *metabolite-2*.

The objective of each cell is to reproduce, which requires essential resources. A cell needs a subset *μ* ^*i*^ ⊆ {1,…,*L*} of *L* metabolites to build a new cell. If $l\in \mu_{l}^{i}$, then the exogenous value $b_{l}^{i}>0$ represents the amount of metabolite *l* that *i* needs in order to reproduce. A species may still transform a metabolite to another even though such metabolite $l\notin \mu_{l}^{i}$ may not be required for reproduction. We assume that one metabolite cannot be substituted by another. Let $x_{l}^{i}\geq 0$ be *i*'s consumption of metabolite *l*. The objective function of *i* is then $u^{i}(x^{i})=\min({\left\{x_{l}^{i}/b_{l}^{i}\right\}}_{l\in \mu_{l}^{i}})$.

An exogenous fraction $c_{l}^{i}\in [0,1]$ of species *i*'s positive net production of metabolite *l* is exported to the shared environment. Let the vector $\omega \in {\mathbb{R}}_{+}^{L}$ indicate the exogenous quantity of each of the *L* metabolites endowed to the medium. The total amount of imports must be less than the total amount of export plus the endowment for each metabolite *l*:

$$k_{l}=\omega_{l}+\sum_{i\in \{i|y_{l}^{i}\geq 0\}}N^{i}c_{l}^{i}y_{l}^{i}-\sum_{i=1}^{I}N^{i}m_{l}^{i}.$$

The stock variable *k*_*l*_ ≥ 0 is the remaining quantity of metabolite *l* that is left in the medium. In the main text we assume the special case where *k*_*l*_ = 0 for expositional purposes.

The biased-access allocation rule in the general form has the following properties. A cell of species *i* has access to each unit of good *l* equal to $(1-c_{l}^{i})/\sum_{j=1}^{I}\left(1-c_{l}^{j}\right)N^{j}$. A cell need not use their full access. Any unused metabolites are distributed to all other cells in a way that is proportional to their access. For example, suppose there are 3 cells and 25 units of the arginine metabolite, with *cell-1* having access to 4 units, *cell-2* having access to 8 units, and *cell-3* having access to 13 units. Suppose *cell-3* only imports 7 units, while the other two cells import their full access. The remaining 6 units of arginine are allocated across the first two cells in proportion to the original access. Since *cell-2* has twice the access as *cell-1*, it gets 4 units of the remaining arginine and *cell-1* gets 2 units. Hence, the import vector for the cells would be *m*_arg_ = (7,12,6). In this paper, we use the biased-access allocation rule because it captures the basic process of diffusion when cells asymmetrically export at different contribution rates. We view this rule as an approximation that expresses key properties of the system: (1) higher contribution rates result in linearly greater exports, all else equal, and (2) the additional active transport inherent in the higher contribution rates results in a reduction in uptake relative to the other species, all else equal. However the allocation rule is a modular component of the model. Other allocation rules can be developed both to exhibit more refined aspects of the environment, and to accommodate features of other environments. For example, environments that limit resource exchange to cells in close physical proximity would require additional allocation constraints.

A *biotic economy* can be summarized by the productive capabilities, resource requirements, population levels, contribution rates of each species, and a vector of endowed metabolites forming the quadruple $\left({\left\{Y^{i},b^{i}\right\}}_{i=1}^{I},{\left\{N^{i}\right\}}_{i=1}^{I},{\left\{c^{i}\right\}}_{i=1}^{I},\omega \right)$. A *biotic allocation* (*x*,*y*,*m*,*k*) = (*x* ^1^,…,*x* ^*I*^,*y*_1_,…,*y* ^*I*^,*m*_1_,…,*m* ^*I*^,*k*), is defined as a consumption, production, and import vector for each cell, and a vector of the stock of metabolites that remain in the shared environment. We now introduce the solution concept.

#### Biotic Equilibrium

Given a biotic economy specified by $\left({\left\{Y^{i},b^{i}\right\}}_{i=1}^{I},{\left\{N^{i}\right\}}_{i=1}^{I},{\left\{c^{i}\right\}}_{i=1}^{I},\omega \right)$, a biotic allocation (x*,y*,m*,k*) constitutes a biotic equilibrium if the following conditions are satisfied

$$(m^{*},x^{*})=\min_{m,x}\sum_{l=1}^{L}\;\sum_{i=1}^{I}\left(-\frac{x_{l}^{i}}{b_{l}^{i}}+\lambda \sum_{j=1}^{I}\left|\frac{m_{l}^{i}}{1-c_{l}^{i}}-\frac{m_{l}^{j}}{1-c_{l}^{j}}\right|\right)\;\text{where}\;\lambda \gg 0$$ (1)

s.t.

$$\text{Utility Maximization}:\;\frac{x_{l}^{i}}{b_{l}^{i}}=\frac{x_{l^{\prime }}^{i}}{b_{l^{\prime }}^{i}}\;\text{for all}\;l,l'\;\text{and}\;i\;\text{when}\;l,l'\in \mu^{i}$$

$$x_{l}^{i}=0\;\text{for all}\;l,l'\;\text{and}\;i\;\text{when}\;l,l'\notin \mu^{i}$$ (2)

$$\text{Budget Constraint}:\;x_{l}^{i}\leq m_{l}^{i}+(1-c_{l}^{i})y_{l}^{i}\;\text{for all}\;l\;\text{and}\;i\;\text{when}\;y_{l}^{i}\geq 0$$

$$x_{l}^{i}\leq m_{l}^{i}+y_{l}^{i}\;\text{for all}\;l\;\text{and}\;i\;\text{when}\;y_{l}^{i}<0$$ (3)

$$\text{Production}:\;y^{i}\in Y^{i}$$ (4)

$$\text{Bounded Imports}:\;m_{l}^{i}\leq {\bar{m}}_{l}^{i}\;\text{for all}\;l\;\text{and}\;i$$ (5)

$$\text{Trade Balance}:\;k_{l}=\omega_{l}+\sum_{i\in \{i|y_{l}^{i}\geq 0\}}N^{i}c_{l}^{i}y_{l}^{i}-\sum_{i=1}^{I}N^{i}m_{l}^{i}\;\text{for all}\;l$$ (6)

These conditions capture two broad principles. The first principle is that cells optimize. Cells maximize their utility, which implies that consumption of metabolites will be given in the ratio indicated in (2). Given that the cell is consuming at the optimal ratio, it maximizes its consumption indicated by the $x_{l}^{i}/b_{l}^{i}$ term in (1). A cell's consumption is limited by the amount of metabolite it produces, the amount it imports, less the amount it exports as indicated by (3). Expression (4) specifies that each cell is bound to produce according to its production set and (5) restricts the maximum amount of import. Eq (6) states that the remaining stock of a metabolite equals the original endowment plus exports minus imports. The second principle is that all cells have biased access to the exports in the environment. Subject to this constraint, the absolute value expression in (1) implements the biased-access allocation rule. When the arbitrary constant *λ* ≫ 0, the biased-access allocation rule supersedes the utility maximization. Thus, for sufficiently large *λ*, this is equivalent to implementing utility maximization subject to the biased-access allocation rule as a constraint. In the 2x2 case the biased access allocation rule reduces to a convenient explicit equation. However, in the general model the biased access allocation rule cannot be easily represented as a set of linear constraints. By placing the term $\left|m_{l}^{i}/(1-c_{l}^{i})-m_{l}^{j}/(1-c_{l}^{j})\right|$ in the objective function, minimization causes the ratio of imports $m_{l}^{i}/m_{l}^{j}$ to approach $\left(1-c_{l}^{i})/(1-c_{l}^{i}\right)$, as described in the main text, subject to satisfying (2)-(6). The constraints that play the most direct role are (2), which constrains imports to be used for growth, and (5) which says that imports may not exceed the physical maximum.

As long as the production set can be specified as a set of linear constraints, this model can be fully solved using linear programming techniques. The resulting biotic equilibrium yields the consumption, production, and imports of each species, as well as the remaining stock of metabolites in the medium. More importantly, the growth rate can be easily computed since it is proportional to utility. The model is depicted in Fig 2 for a 2-species 2-metabolite case.

This economy bears similarities and some striking differences to most standard textbook general equilibrium economies. Essentially, BGET is an archipelago-extension of the Robinson Crusoe model with redistribution [33]. The model is isomorphic to a collection of Robinson Crusoe economies, in which a central authority taxes and redistributes across the agents according to the biased-access allocation rule. Thus, the concept of a market-clearing price plays a less important role in this economy since each autarkic agent faces its own price vector. There does not exist a single market-clearing price. Instead, exchange is driven by the exogenous contributions and biased-access allocation rule.

Biotic equilibrium specifies the growth rate of all species at a single period in time. As the economy grows over time, the population ratios and stock of goods may change thereby changing the biotic equilibrium. We focus on steady state population vectors.

#### Steady State

A population vector *N* = (1,*N* ^2*^,…,*N* ^I*^) constitutes a steady state in the biotic economy specified by $\left({\left\{Y^{i},b^{i}\right\}}_{i=1}^{I},N^{*},{\left\{c^{i}\right\}}_{i=1}^{I},\omega \right)$, if the utility vector u* = (*u* ^1*^,…,*u* ^I*^) generated from the biotic equilibrium (x*,y*,m*,k*) is u* = *r*⋅(1,…,1) where *r* ≥ 0 is a scalar.

A population vector is said to be in steady state if the resulting biotic equilibrium results in equal growth rates for all species. This is a steady state of population ratios but not of population levels.

An unstable steady state is a steady state for which small perturbations in the population ratio lead the ratio away from the steady state in the long run. A stable steady state (SSS) is a steady state for which small perturbations in the population ratio have no effect on the long-run population ratio. Let *v*(*N*) be the indirect utility function that takes the utility values *u*(x*) resulting from the biotic equilibrium.

#### Steady Stable State

Let the vector $\delta^{i}\in {\mathbb{R}}^{L}$ take the value *δ* > 0 for the *i*th element and 0 for all other elements. A steady-state N** in the biotic economy $\left({\left\{Y^{i},b^{i}\right\}}_{i=1}^{I},N^{**},{\left\{c^{i}\right\}}_{i=1}^{I},\omega \right)$ is stable if there exists a $\bar{\delta^{i}}>0$ such that *v* ^*i*^(*N* + *δ* ^*i*^) < *v* ^*j*^(*N* + *δ* ^*i*^) for all $\delta^{i}\leq \bar{\delta^{i}}$ for all *j* and *i*.

This statement implies that a steady state is stable if a sufficiently small increase to population *i* leads its growth rate to be lower than the growth rate of all other species.

### Strains and Growth Media

*E*. *coli* strains used were derived from EcNR1 [44], which contains an integrated λ-Red recombineering system. The *ΔR* and *ΔF* strains were generated as described previously [13]. Fluorescent genes for mCherry and sfGFP were inserted into the neutral *galK* genomic locus of *ΔR* and *ΔF* respectively by recombineering. Genes *yddG* and *argO* for amino acid export and sfGFP were cloned into separate pZA vectors from Expressys under pL-tetO regulation. Translation initiation sequences driving a range of exporter expression were selected from [45] and cloned into each vector.

### Co-culture Quantification

Cells grown into late exponential phase in LB-Lennox were first washed in M9 minimal media (6 g/L Na2HPO4, 3 g/L KH2PO4, 1 g/L NH4Cl, 0.5 g/L NaCl, 1mM MgSO4-7H2O, 0.083 nM thiamine, 0.25 mg/ml biotin, 0.2% glucose). Co-culture kinetic assays were performed by inoculating a 1:1 ratio of each strain at 10^7^ cells/ml into 200ul of M9 media containing 1ug/ml aTc to induce the pL-tetO promoter. Kinetic growth assays were done on a plate reader in 96-well format at 30°C. Cells were fixed in 1x PBS 1% PFA solution after 24-hours. Cell concentrations and growth rates were determined based on spectrophotometer OD600 readings. Relative number of mCherry and sfGFP cells were quantified via flow cytometry and normalized to the highest mean FITC value to determine the relative abundance of each strain in the population. All experiments were done in three biological replicates.

## Supporting Information

S1 Appendix Understanding the Stable Contribution-Space. Explains the intuition behind comparative advantage and the stable steady states.
(PDF)

S1 Script Calculates SSS in the 2x2 Model. Mathematica script used in the construction of Fig 5.
(NB) Click here for additional data file.
