# Supplementary material for: An Economic Framework of Microbial Trade
Source: PLoS One. 2015 Jul 29;10(7):e0132907. doi: 10.1371/journal.pone.0132907 (PMC4519184; doi:10.1371/journal.pone.0132907)
Supplement: S1 Appendix — Explains the intuition behind comparative advantage and the stable steady states. (PDF) [file pone.0132907.s001.pdf]

SUPPORTING INFORMATION – S1 – APPENDIX

An Economic Framework of Microbial Trade

Joshua Tasoff*, Michael T. Mee†, Harris H. Wang§

* Department of Economics, Claremont Graduate University, Claremont, CA 91711,

† Department of Biomedical Engineering, Boston University, Boston, MA 02215,

§ Columbia University, Department of Systems Biology, New York, NY 10032

UNDERSTANDING THE STABLE CONTRIBUTION-SPACE

Comparative Advantage is Necessary for Mutualistic Exchange. In biotic equilibrium all cells optimize. Thus for every unit of good l exported, the only way for a cell to increase utility is to receive imports that on net expand the consumption set, that is, are energetically more valuable for that species than the export. In other words, for every unit of good l exported, the cell must receive $a^i_{l'}/a^i_l$ units of good l' to increase its utility. Let z^i_l be the total amount of good l lost from trade by species i. Now consider a single cell of each species i and j. For i to have increased utility after losing z^i_l it must receive at least $(a^i_{l'}/a^i_l)z^i_l$ units of l'. Likewise for j to have increased utility after losing $z^j_{l'}$ it must receive at least $(a^j_l/a^j_{l'})z^j_{l'}$ units of l. Thus we have that $z^j_{l'} \geq (a^i_{l'}/a^i_l)z^i_l$ and $z^i_l \geq (a^j_l/a^j_{l'})z^j_{l'}$ therefore $a^j_{l'}/a^j_l \geq a^i_{l'}/a^i_l$. This is the definition of comparative advantage.

The Two Benefits of Giving. As Figure 5e demonstrates, when there is comparative advantage and species-j has a positive contribution rate, then it is optimal from a growth-maximizing perspective for species-i to export as well. A species that has a zero contribution rate actually grows more slowly than one that has a positive contribution rate. There are two mechanisms driving this result. The first is the direct effect whereby sending metabolites to the trading partner causes the partner to specialize production more in its comparative-advantage metabolite (i.e. the metabolite that it exports) thereby causing the trading partner to export more. When there is comparative advantage, the value in terms of glucose requirements of all imported metabolites can exceed the value of all exported metabolites. The second mechanism manifests in the dynamics. The trading partner is a source of necessary metabolites. By exporting metabolites to the partner to help the partner grow, the species obtains more trading partners in the future and consequently more imports in the future.

Adjustments in the Population Ratio Maintain Coexistence. Figure 4 depicts the logic behind a SSS. When the population ratio is perturbed upward from N^{**} (*species-2* population over *species-1* population) the growth rate of *species-1* increases and the growth rate of *species-2* decreases,

leading the population ratio to converge back to N^{**}. The increase in the population ratio leads to more trading partners for *species-1* and less trading partners for *species-2*, causing *species-1*'s growth rate to exceed that of *species-2*. A similar logic is true for a downward perturbation. A change in a species' contribution rate changes the shape of the growth curves in Figure 4, possibly inducing a new steady state.

This adjustment-mechanism through the population ratio allows for species-coexistence to be robust to perturbations in the contribution rates as well. In contrast to classical chemostat models, our biotic general equilibrium theory has an equilibrating mechanism that allows two species to co-exist at many possible contribution levels. Mathematically this means that the stable contribution-space (Figure 5a-c) has greater than zero measure – that is for a given contribution level of species-j there are often many contribution levels of species-i that induce SSS (i.e. graphically this means that the stable contribution-space has area; it is thicker than a curve). The intuition for why this is the case is that there is a cost to having a higher contribution rate and a benefit to having a lower relative abundance. When a species' contribution rate increases, consumption of that metabolite decreases. This has the direct effect of reducing the growth rate of this species, which decreases its relative abundance in the population. On the other hand, a lower relative abundance leads to more trading partners from whom to import metabolites. More trading partners may increase the growth rate of the species, as long as there is comparative advantage. The benefit of more imports at lower relative abundance counteracts the increased burden of having a higher contribution rate. If a steady state exists, this lower relative abundance fully offsets the initial increase in the contribution rate leading to a new steady-state population ratio and new steady-state growth rate. In this way, a community of two species can coexist growing stably at the same rate.

Shape of the Stable Contribution-Space. The shape of the stable contribution-space can take on many forms. Figure 5a-c illustrates three similar and symmetric examples. In general, we find that when contribution rates are very high for one species and low for the other, SSS does not exist. For these parameters, one species is heavily burdened by over-sharing, while the other species contributes little. Consequently, the low-contribution species will grow faster. Consider a contour line in the upper left half of one of the panels in Figure 5a-c. As *species-1* increases its contribution rate, the contribution rate of *species-2* must also increase in order to maintain the same population ratio in SSS. Otherwise the population ratio begins to favor *species-2*. As we follow the contour line, we eventually encounter a kink. Here, no additional contribution of *species-2* is required to maintain the population ratio. In this region an increase in *species-1*'s contribution rate has no effect; *species-2*'s limiting resource is metabolite 2 and so *species-1* reabsorbs all of its export. Since the example is symmetric, a similar logic applies to the contours in the lower right half of each of the panels.

At higher contribution rates the contour lines in Figures 5a-b produce apparent asymmetries.

The contour lines are symmetric, but there are two SSS and the surface displayed is the higher SSS and is superimposed on the lower SSS. In this region the initial population ratio will determine the SSS. A high initial population ratio leads to the SSS in which metabolite 1 is the limiting metabolite for both species, and the reverse is true for a low initial population ratio. As Figure 5c shows, multiple SSS do not always exist. The figures also show that SSS may not exist at mutually high contribution rates. In these regions a cell's export becomes its limiting factor due to the high contribution rate. Growth is then determined largely by the cell's ability to re-import its export which is bounded by $\bar{m}_i^i$. When contribution rates are very high, the driver of growth is based heavily on this parameter. The population ratio as an equilibrating mechanism described above is less effective and unable to generate stability.
